# Navigating Collective Bargaining Barriers to the Implementation of AI Scribe Technology Among Ambulatory Advanced Practice Providers: Mixed Methods Quality Improvement Study

**DOI:** 10.2196/92364

**Published:** 2026-09-17

**Authors:** Aaron Wright, Danise Seaters, Christi DeLemos

**Affiliations:** 1 University of California Davis Medical Center Sacramento, CA United States

**Keywords:** AI scribe, advanced practice providers, collective bargaining, medical informatics, documentation efficiency, ambulatory care, artificial intelligence, AI

## Abstract

**Background:**

Advanced practice providers (APPs) face rising documentation demands driven by increased productivity pressures, contributing to burnout and reduced time with patients. AI scribes may reduce documentation time and improve clinician well-being and patient interaction. Labor unions have expressed concerns about AI in health care affecting job security, safety, ethics, and governance. Little is known about the deployment of AI scribes for APPs in a union environment.

**Objective:**

This project aimed to describe the process, challenges, and outcomes of implementing an AI scribe for APPs within a unionized ambulatory academic setting, focusing on collective bargaining considerations and using a pilot project to help address APP union concerns.

**Methods:**

Following formal notices to labor unions and a meet-and-confer process consistent with California public employer obligations, we conducted an 11-week (55-workday) pilot (June 16 to August 31, 2025) of the Abridge AI scribe among 15 primary care APPs (n=12 nurse practitioners and n=3 physician assistants). Training modules covered consent, privacy, and documentation verification. We tracked scribe use, percentage of AI notes kept, time spent on notes, and same-day encounter closures; results were reported to stakeholders. Pilot metrics were compared with those of our organization’s nonpilot APPs, and additional postpilot survey data were obtained from the APPs by the vendor. This information, along with the required training and patient and health care provider protections, was used to validate this technology to the labor unions.

**Results:**

Across 8100 APP encounters, the AI scribe was used in 5403 (66.7%) notes. Individual use ranged from 30% (54/179) to 89% (501/563; SD 16.9%). The average percentage of AI-generated note content retained by the APP was 78% (SD 18.2%; range 20%-93%). The time spent in the note writer for the pilot group was a mean 6 (SD 2.2; median 6, IQR 5.0-8.0) minutes vs a mean 14 (SD 11.2; median 11, IQR 8.4-18.5) minutes for nonpilot APPs. Same-day encounter closure in the pilot group was 90% (7290/8100; SD 9.3%) vs 72% (23,682/32,983; SD 30.0%) in the nonpilot group. Postpilot feedback from the pilot group showed positive APP sentiment. No postpilot formal or informal concerns were raised, and the labor unions agreed to widespread implementation of AI scribe technology across the organization for our ambulatory APPs.

**Conclusions:**

AI scribe implementation among unionized APPs was feasible when paired with structured labor engagement, pilot-testing, privacy safeguards, and optional adoption. The pilot yielded descriptive information showing a difference between pilot and nonpilot AI scribe users, with limited generalizability beyond the context of labor union engagement. The pilot’s efficiency differences and documentation timeliness align with existing published data, which gave confidence in the labor unions’ adoption of the technology. Future work will examine variability in use and effort reduction across APPs.

## Introduction

There is a dearth of literature that analyzes AI scribe adoption specifically by advanced practice providers (APPs) within unionized ambulatory environments. APPs are twice as likely as physicians to be represented under collective bargaining, and the logistical impacts of those labor unions’ stances on AI scribe technology are not well known [1].

Our organization explored the novel challenges of deploying an AI scribe within a unionized APP environment and offers a potentially scalable framework for advancing implementation.

In our health system, staff physicians are classified as unrepresented academic appointees or nonrepresented management. Ambulatory physician documentation burden often includes over 2 hours of work for every 8 hours of patient encounters; frequently, this is work that is conducted outside of regular clinic hours [2].

Dissimilar to physician cultural expectations, our APP union contracts outline specific working conditions with grievance procedures; shift and scheduling protections including overtime pay; and mechanisms for addressing clinical practice issues, equipment, and professional standards. Furthermore, comparisons between physician and APP workflows are challenging because of variable task allocation, limited scopes of practice, and different compensation models.

In the United States, APPs face increasing expectations to meet patient access demands and justify their costs to their organization [3,4]. Increased documentation burden shifts work time attention away from patient-centered care and increases the risk of provider burnout [5]. Published data demonstrate that AI scribe tools can reduce documentation time, improve work-life balance, and maintain documentation compliance [6,7]. Prior investigations at our own institution have shown that patients are open and accepting of AI scribe technology, ascribing these feelings to enhanced focus by the clinician on the patient during the encounter [8]. Our intention was to expand this technology to include APP visits, allowing them to have the same opportunity to focus on the patient.

Despite the celebrated benefits of AI technologies in health care, many ethical and regulatory challenges exist, including patient safety and privacy concerns [9]. Collective bargaining organizations that represent health care workers also pose questions regarding the negative effects of technology on patients and health care workers. A 2024 survey by the National Nurses United labor union associated with the California Nurses Association argued that AI technology “often contradicts and undermines nurses’ own clinical judgment and threatens patient safety” [10]. In that press release, National Nurses United stated that the survey results “underscore the urgent need for stricter regulation and greater input from nurses and health care staff on whether and how AI is deployed” [10].

After piloting and implementing AI scribe technology in the prior fiscal year for our ambulatory physicians, who are not represented by a labor union, our organization wanted to expand the same efficiency tools to our APPs. Because this was considered a change in working conditions for the APPs, a formal process of union engagement was undertaken and used to implement the AI scribe.

Our institution’s contract with the California Nurses Association also includes language that the use of technology should not limit clinical judgment or care implementation. Furthermore, the union contract terms support the central role that represented staff play in evaluating and validating the new technology [11]. Extrapolation of physician experience with AI scribes, either published or institutional, would not satisfy our labor groups’ expectations for proving the appropriateness of the technology for their APP members.

## Methods

### Ethical Considerations

This pilot project was conducted as a voluntary performance improvement process to evaluate the implementation of an AI clinical documentation tool within routine clinical practice by a small subset of our ambulatory APPs. This process replicated a previously implemented physician pilot in which no institutional review board (IRB) approval was sought. Within the organization, performance improvement or quality improvement projects do not require IRB approval if their sole purpose is to evaluate, improve, or maintain the quality of a local health care service or operational process. Participation by APPs was optional and not required as a condition of employment. The pilot was initiated to assess workflow impact and address labor union concerns related to the adoption of new technology rather than to obtain generalizable knowledge. This work did not meet the definition of human subject research, and therefore, IRB review was not sought by the pilot’s governance team, which comprised legal counsel, physician leadership, and other health system leaders. No patients were recruited for research purposes, and patient care was not altered beyond routine documentation processes that were already in place for physician workflows within the organization. Use of the AI scribe technology during the pilot was governed by an existing organizational oversight committee, which provided review and guidance regarding implementation, consent, privacy, and responsible use. All data analyzed were derived from standard operational metrics and were deidentified prior to analysis.

### Data Reporting and Analysis

The 15 APPs in primary care, who are our largest ambulatory homogeneous APP team and primarily manage established and follow-up patient types, were identified in partnership with the labor unions as a viable group for initially assessing and addressing the labor concerns about the use of the technology. There were several reasons for this selection. The use of primary care as a care area followed our physician specialty pattern of AI scribe implementation and created an opportunity to evaluate a large number of APP-patient interactions in a short period. Our primary care APPs also have the largest geographic regional separation in the health system, which the unions felt may help elicit a broader range of APP concerns.

The deidentified pilot and nonpilot data were obtained from Epic Signal data, and definitions were applied consistently across clinicians and the reporting period of June 16, 2025, to August 31, 2025. The pilot APP data were compared against those from 98 nonpilot APPs (n=77, 78.6% nurse practitioners; n=21, 21.4% physician assistants) in the institution working in pediatrics, women’s health, and medical and surgical specialty clinics during the same time frame. As the comparison was for descriptive purposes for the union, no rigorous analysis was performed by our organization or requested of the vendor.

Abridge reported an operational metric referred to as “effort reduction,” which was presented to our organization as the percentage of AI-generated note content retained by the APP after review and editing. Higher percentages therefore indicate that a larger proportion of the AI-generated documentation was preserved in the final note, whereas lower percentages indicate more substantial editing by the APP. Abridge did not provide details regarding the specific types or locations of edits performed within the notes, which limits interpretation of this metric.

Same-day encounter closure was defined as completion and final signing of the clinical note on the same calendar day as the patient encounter. Time in the note writer represented the cumulative duration of active clinician interaction with the note-authoring interface.

As part of the pilot process, the vendor invited the pilot APP group to complete a proprietary qualitative-type survey, with questions blinded to our organization, to elicit the APPs’ experience with the AI scribe. The vendor-administered and analyzed survey information was reported to our institution by the vendor. No independent reanalysis of the survey data was conducted by our organization, nor was any survey conducted with the nonpilot group. Furthermore, the questions and validity of the survey were not scrutinized.

### Meet and Confer

In 2024, our organization piloted a limited number of physician AI scribe licenses through the same AI scribe vendor. The positive physician sentiment spurred APP interest in the scribe for their own practices. Following guidance from our employee labor relations team, our office submitted a formal notice to the unions representing our APPs that our institution intended to expand and deploy the AI scribe technology to APPs. Under our agreements, the unions required 30 days’ notice prior to the change in any working conditions.

This notice prompted requests for “meet and confer” meetings with the unions to discuss what the representatives of the labor force considered to be a change in working conditions. This process is a legal obligation for California public higher education institutions and their unionized employees under the Higher Education Employer-Employee Relations Act (HEERA), where public employers must negotiate in good faith over wages, hours, and working conditions before implementing changes [12]. These meetings involve a dialogue meant to exchange information and proposals to reach agreements consistent with an existing labor contract.

During these meetings, a range of concerns were raised by the union leaders, APP union representatives, and members of the APP labor force. Common themes included patient privacy and the use of the audio recordings for worker surveillance and discipline. There were general concerns about the provision of workforce training and availability of health system–supplied devices. The unions also raised concerns that the technology would be used to replace APP clinical decision-making, self-generate diagnoses, and formulate treatment plans.

### AI Legal Boundaries

The unions’ primary concerns were addressed through contractual agreements from the vendor that its product aligns with HIPAA (Health Insurance Portability and Accountability Act) standards [13]. This was further supported with the language found in California’s Confidentiality of Medical Information Act, which restricts use and disclosure of medical information [14].

Our institution and the vendor also follow the California Invasion of Privacy Act, which requires consent from all parties before recording private conversations. For AI scribes to record sessions, explicit consent must be obtained from everyone present for the clinical encounter [15].

Additionally, according to state law, select sensitive and marginalized patient populations are also excluded from the use of an AI scribe. Medical data related to abortion, contraception, fertility, and gender-affirming care are considered sensitive and require electronic health record systems to implement privacy controls and limit data sharing [14]. Electronic health record vendors such as Abridge that offer such data services are subject to the Confidentiality of Medical Information Act strict storage, disclosure, and use protections. All users of the AI scribe technology received formal training from the vendor and our organization on the use of the technology being forbidden with these patient populations and the need to stop the recording should any of these issues be brought up during a patient encounter.

The unions’ concerns regarding AI-generated diagnoses and health care plans were assuaged by describing the functional limitations of the product, which does not self-generate diagnoses or plans. Additionally, state law prohibits anyone other than licensed professionals, including AI, from diagnosing and treating patients [16]. The union representatives were also shown that the AI scribe’s sole function is to transcribe conversations into a progress note; all diagnoses, subsequent orders, and treatment plans remain the purview of the APP. Mandatory training was required to ensure that all providers were educated on the compliant use of the AI scribe, constraints of the technology, and patient consent process.

### Concerns Related to Employee Management

Prior to being able to access the AI scribe technology, all physicians and APPs were required by our health system to complete an online training module. The module outlines the need and process for obtaining patient consent, the logistics of using the AI scribe, and the regulatory restrictions of its use. It also provides guidance regarding verifying the accuracy of the clinical documentation and a means to provide feedback to the vendor if the scribe is inaccurate. These training modules, containing content from the vendor and the health system, satisfied the unions’ concerns. Compliance with these rules and regulations is monitored by our organization’s health information management team and compliance department.

The ultimate goal of the AI scribe expansion to APPs was to reduce administrative tasks and improve efficiency, not to initiate APP disciplinary proceedings. The audio recordings are saved for 30 days before being deleted from the system. While it is conceivable that the recording could be used in a possible disciplinary process, the health system was also able to convey to the unions that the recording could also be used to vindicate an APP falsely accused of unprofessional behavior by staff or patients. The formal notification to the unions also further clarified our health system’s intention that the recordings generated by the AI scribe would not be used for the purposes of employee monitoring except in the process of due diligence related to policy violations or instances of system misuse. Through our organization’s labor consultant, the unions were formally notified that “It is imperative to emphasize that the recordings generated by this application will not be employed for the purpose of employee monitoring. However, in the event of policy violations or instances of system misuse coming to our attention, we are unequivocally committed to addressing such matters with the utmost diligence and in strict accordance with established policies and procedures.”

The use of an APP’s own device in our institution is optional, and our health system supplied tablet-type devices to employees using the scribe. Additionally, it was reinforced that the use of the new technology was optional and not a requirement of the APPs’ conditions of employment. If, for any reason, an APP did not want to use the AI scribe, they were under no obligation to do so.

### APP Pilot Program

The unions and the health system agreed to an 11-week (55-workday) pilot study with a select group of APPs to assess the AI scribe as part of their responsibility to evaluate new technology that could impact their members and lay the groundwork for discussing future broader use of the technology by APPs. Our institution’s analytics oversight committee, comprising physician leadership, legal counsel, and other health system leaders, was also engaged to approve and monitor the expansion.

During the pilot, 3 provider feedback sessions were held to provide training, assistance, and elicit feedback on the AI scribe tool. Weekly emails were also sent to APP leadership to identify issues warranting collective bargaining considerations and general progress of the pilot. The training sessions also reinforced the need to follow regulatory and policy expectations regarding visit types that are not allowed to be conducted with an AI scribe.

The pilot tracked 4 data points to report back to the union as part of the evaluation of this technology. These were the total number of notes written by the AI scribe, the average percentage of the AI note kept by the APP, the time spent on notes, and the percentage of appointments closed on the same day of the encounter. Pilot and nonpilot APP data were reported to the analytics oversight committee and APP leadership, and a postpilot survey of the involved APPs was conducted by the vendor.

## Results

A total of 8100 APP-patient visits were completed during the pilot window from June 16, 2025, to August 31, 2025, fewer than would normally be possible due to lower seasonal booking rates and APP vacations. During the same time frame, 32,983 patient visits were received by APPs not participating in the pilot. The AI scribe was used in 66.7% (5403/8100) of the pilot group encounters. Neither our institution nor the vendor captured data on the rationale for AI scribe nonuse. No cases of APP noncompliance with the regulatory or policy expectations for AI scribe use were reported during the pilot by our health system.

The use of the AI scribe by each APP ranged from 30% (54/179) to 89% (501/563) of encounters (mean 66%, SD 16.9%; median 68%; IQR 57%-77%). The percentage of AI-generated note content retained by the APPs, reported by the vendor as its “effort reduction” metric, averaged 78% (SD 18.2%; median 86%; IQR 77%-88%), with a range of 20% to 93%. The average time an APP in the pilot spent in the note writer section of the patient encounter was 6 (SD 2.2; median 6, IQR 5.0-8.0) minutes, ranging from 4 to 11 minutes per APP. Nonpilot ambulatory APPs in the institution working during the same time frame averaged 14 (SD 11.2) minutes per encounter note, ranging from 1 to 72 (median 11, IQR 8.4-18.5) minutes. The percentage of encounters closed on the same day as the visit in the pilot APP group was 90% (7290/8100; SD 9.3%; median 92%, range 62%-100%, IQR 87%-96%) during the pilot time frame compared with 72% (23,682/32,983; SD 30.0%; median 84%, range 0%-100%, IQR 50.0%-96.6%) in the nonpilot group. A summary comparison of pilot and nonpilot APP performance metrics are shown in Table 1.

**Table 1:** Quantitative comparison of ambulatory advanced practice providers (APPs) participating in an 11-week quality improvement AI scribe pilot vs nonpilot APPs at an academic health system in Sacramento, California^a^.

			Pilot APP group (n=15)		Nonpilot APP group (n=98)
Total encounters, n			8100		32,983
**Cohort composition, n (%)**					
	Nurse practitioners	12 (80)		77 (78.6)	
	Physician assistants	3 (20)		21 (21.4)	
Encounters using the AI scribe, n/N (%)			5403/8100 (66.7)		—^b^
Percentage of AI-generated note kept by the APP—Abridge “effort reduction” metric (%), mean (SD; IQR)			78 (18.2; 77-88)		—
Percentage of AI-generated note kept by the APP—Abridge “effort reduction” metric (%), median (range)			86 (20-93)		—
Time spent by the APP in the note writer (min), mean (SD; IQR)			6 (2.2; 5.0-8.0)		14 (11.2; 8.4-18.5)
Time spent by the APP in the note writer (min), median (range)			6 (4-11)		11 (1-72)
Encounters closed on the same day (%), mean (SD; IQR)			90 (9.3; 87-96)		72 (30.0; 50.0-96.6)
Encounters closed on the same day (%), median (range)			92 (62-100)		84 (0-100)

^a^The pilot cohort included 15 primary care APPs (n=12, 80% nurse practitioners and n=3, 20% physician assistants), and the comparison cohort included 98 nonpilot APPs (n=77, 78.6% nurse practitioners and n=21, 21.4% physician assistants) practicing in pediatrics, women’s health, and medical and surgical specialty clinics. Metrics were collected during the observation period from June 16, 2025, to August 31, 2025.

^b^Not applicable.

During the APP pilot feedback sessions, training and assistance were provided to and feedback was elicited from the pilot group. APPs who participated in the sessions reported favorable changes in efficiency and organization, especially for complex patient visits, citing appreciation for a bullet-point history of present illness format and minimal editing needs. Technical questions from the APPs focused on how to adjust their note template types and how to customize preferences and settings. The APPs reported mixed levels of comfort with using the technology, mostly ascribed to greater familiarity with legacy documentation practices. No complaints or rejections of the technology were raised by the pilot group.

In the planning phase, during the 3 feedback sessions throughout the pilot, and after the completion of the pilot, the labor unions, representatives, and APPs were engaged to discuss any concerns with moving the AI scribe forward. These engagements provided opportunities for the impacted parties to raise concerns verbally or in writing through established channels. There were no concerns shared verbally or expressed in writing by the labor unions. The absence of union concerns was operationalized and recorded as the lack of submitted grievances, requests for bargaining, requests for additional information, or documented objections across these formal and any informal processes. These engagements included opportunities to discuss items such as privacy issues or any ongoing concerns regarding audio recordings being used for disciplinary action. Beyond the concerns raised during the initial meet-and-confer process about productivity monitoring or performance evaluation, neither the union nor any employee raised any issues of substantive concern.

In the proprietary postpilot survey conducted by the vendor, the tenor of the open-ended input elicited from the pilot APP group was generally positive. The vendor reported that 60% (9/15) of the APPs described needing less time for documentation and felt that the scribe helped with note organization and improved detail capture. In total, 20% (3/15) of the APPs provided critiques regarding connectivity and server issues as well as delays in note processing speed from the time of the visit ending until the note was available for review. A total of 93.3% (14/15) of the APPs reported that they felt they had the ability to provide undivided attention to their patients after using the scribe; 6.7% (1/15) reported neutral feelings. In total, 86.7% (13/15) felt that the technology improved their work satisfaction; 13.3% (2/15) reported neutral feelings.

The pilot APPs offered nonspecific critiques to the vendor when asked how to make the technology more valuable. One APP cited a need for improvement in the physical examination section of the clinical note without elaboration. There were also 2 concerns raised by 2 different APPs regarding the formatting style and organization of the history of present illness section without further explanation.

The results of the pilot were subsequently shared broadly across the 5 health system campuses whose APPs are represented by the same unions but who operate independently. The unions were again formally noticed of our intent to expand access to the AI scribe to the entire ambulatory APP workforce under the same conditions of training and regulatory restriction. No additional information or meetings were requested by the labor unions, and a postpilot expansion of the AI scribe for APPs was implemented across all ambulatory clinic locations after the conclusion of the pilot.

## Discussion

### Principal Findings

The principal findings of this pilot demonstrated that AI scribes can be implemented among unionized APPs when partnered with structured labor engagement, privacy safeguards, and optional adoption. The implementation of a pilot program enhanced trust and transparency and, ultimately, helped with adoption of the AI scribe technology by the labor unions. The novelty of the pilot and its structured meetings and trainings allowed for greater input from the represented APPs on whether and how the AI scribe is deployed. This process gave the employees and unions timely access to those in charge of controlling the technology’s implementation.

Union involvement early in the pilot design phase ensured that APP concerns, particularly regarding job security, scope of practice, workload redistribution, and data governance, were explicitly addressed. This collaborative governance approach reduced skepticism and was helpful in overcoming the union’s own published concern that AI limits clinical judgment and risks patient safety.

This performance improvement process provided an intermediate step that helped meet the APPs’ own requests for documentation assistance and provided a platform to evaluate organization-wide expansion. The postpilot comments from the APPs suggested that the AI scribe was perceived by the APPs as helpful with their documentation burden. For our organization, the pilot process offered a potentially scalable solution to future technology deployments with APPs when managing labor-related change management issues.

### Comparison to Prior Work

Prior investigations have consistently demonstrated that AI scribes can reduce documentation burden, improve clinician satisfaction, and enhance patient-clinician interaction by allowing providers to focus more fully on the encounter [5-8]. However, much of this prior work has focused primarily on technological performance, usability outcomes, and time savings metrics, with comparatively limited attention to the organizational and labor dynamics that impact adoption and sustainability.

Barriers such as concerns about data privacy, accuracy, workflow disruption, and potential surveillance or productivity monitoring have been previously documented [9,10]. In contrast, our experience demonstrates that formal partnership with unions introduces a distinct and highly effective mechanism for addressing these barriers. By involving workforce representation directly with governance and decision-making processes, we were able to operationalize trust building in a way that extends beyond traditional stakeholder engagement models.

The partnership model described in this paper offers a pragmatic pathway for testing the new technology while integrating labor regulations. This contrasts with prior published descriptions where governance is emphasized but implementation of oversight is less defined [7,9].

### Limitations

While the observed efficiency differences align with physician-focused AI scribe literature and lay the groundwork for extending these findings to APPs, a group underrepresented in prior studies, there are several significant limitations when making any comparative or generalization claim [5,7,17]. Unknown at the time of this pilot was the likelihood that there were other unknown concomitant initiatives in the institution that may have also impacted documentation workflows. Given the nonrigorous methodology of this pilot, these findings should be interpreted with significant caution.

Notably, the purposeful sampling of the pilot APP group introduced a selection bias, and their comparison against predominantly non–primary care APPs is significantly limiting. The other nonpilot specialties who use APPs may have different documentation needs, patient encounter cadences, and visit volume and type expectations. Additionally, the clinical specialties and patient needs of the 2 groups may be vastly different. This clinical mismatch of pilot vs nonpilot APPs is a major limitation of our experience. Further compounding these limitations, there were no prior performance levels used as benchmark comparisons within either group.

Limiting the operational effectiveness of this work, no patient-level outcomes, satisfaction indicators, or documentation quality checks were assessed during the pilot. No assessments of differences in performances within each participating APP were performed. Analysis of the type of edits performed by the APPs in their AI-generated notes and in what sections was not performed, which is a significant hindrance to understanding any benefit to the technology.

While our health system reported no breaches of compliance with state regulations during the pilot regarding AI scribe use in prohibited populations, we performed no independent audits to assess the instances of nonuse of the scribe. No process was in place, nor was any delineation made, to determine whether nonuse was related to compliance reasons, patient consent, or APP preference. This was a major limitation of our process.

The survey responses provided by the represented staff along with the observed efficiency differences were sufficient to advance the full implementation of the scribe with the unions’ agreement. However, survey analyses were conducted by the vendor, and the research team received only aggregated results, which limited independent verification and only allowed for thematic analysis. As the vendor has a financial interest in the technology’s success, interpreting proprietary survey data as evidence of quality improvement or a measure of clinician response to the technology should be viewed as a severe limitation. In addition, no surveying of nonpilot APPs was performed as a comparison.

### Future Directions

Future quality improvement efforts will focus on understanding variability in AI scribe use across APPs and evaluating the impact of AI-assisted documentation on patient satisfaction, clinician well-being, documentation quality, and vendor-reported effort reduction. Additional work may also explore specialty-specific implementation considerations and factors associated with sustained adoption.

### Conclusions

Our experience with implementing the AI scribe with APPs should only be viewed as an example process to achieve an institutional goal, not as reinforcing existing AI efficiency studies or validating its use. Our pilot process serves to highlight a negotiated trust between management and labor rather than elimination of risk.

Public statements from collective bargaining organizations related to technology changes for these represented workforces highlight barriers and potential delays to implementation. Organized labor positions also echo assertions made by other authors that robust regulatory frameworks should be implemented to define responsibility and accountability for multiple areas of concern with this evolving technology [9,17].

In this instance, our collective bargaining organizations have taken a cautious stance toward the adoption of AI scribe technology due to patient privacy, accuracy, uncertainty regarding workload expectations, and surveillance concerns. Open dialogue with the labor unions and their members, our emphasis that AI should augment and not replace our clinicians, and the use of a pilot program all aligned with our existing state regulatory protections to move the technology forward at our institution. Piloting the AI scribe with a select group of APPs significantly limited the creation of generalizable scientific knowledge, but it gave us an opportunity for a structured dialogue with the labor unions and a way for them to feel engaged in the evaluation of the technology’s impact on their members.

## Data Availability

The datasets generated or analyzed during this study are not publicly available due to privacy and institutional policy, but deidentified data are available from the corresponding author on reasonable request.

## Abbreviations

APP: advanced practice provider
HIPAA: Health Insurance Portability and Accountability Act
IRB: institutional review board
